# Phospholipase A2 regulates autophagy in gouty arthritis: proteomic and metabolomic studies

**DOI:** 10.1186/s12967-023-04114-6

**Published:** 2023-04-17

**Authors:** Weili Fu, Minghao Ge, Jian Li

**Affiliations:** Department of Orthopedics, Orthopedic Research Institute, West China Hospital, Sichuan University, Chengdu, 610041 China

**Keywords:** Gouty arthritis, Proteomic, Autophagy, Metabolomic, Lysosome

## Abstract

**Background:**

Acute gouty arthritis is inflammatory joint arthritis. Gouty arthritis (GA) involves multiple pathological processes. Deposition of joints by monosodium urate (MSU) crystals has been shown to play a critical role in the injury process. Due to the different effects of MSU stimulation on the joints, the exact changes in the synovial fluid are unknown. We want to explore the changes in proteins and metabolites in the joints of gouty arthritis. Regulating various functional substances in the joint can reduce inflammation and pain symptoms.

**Methods:**

10 patients with gouty knee arthritis and 10 normal controls were selected from clinical, surgical cases. The biological function of the metabolome was assessed by co-expression network analysis. A molecular network based on metabolomic and proteomic data was constructed to study critical molecules. The fundamental molecular changes in the relevant pathways were then verified by western blot.

**Results:**

Proteomic analysis showed that the expressions of proteases Cathepsin B, Cathepsin D, Cathepsin G, and Cathepsin S in synovial fluid patients with gouty arthritis were significantly increased. Enrichment analysis showed a positive correlation between lysosomal and clinical inflammatory cell shape changes. Untargeted metabolomic analysis revealed that lipids and lipoids accumulate, inhibit autophagic flux, and modulate inflammation and immunity in gouty arthritis patients. It was determined that the accumulation of lipid substances such as phospholipase A2 led to the imbalanced state of the autophagy-lysosome complex, and the differentially expressed metabolites of Stearoylcarnitine, Tetradecanoylcarnitine, Palmitoylcarnitine were identified (|log2 fold change|> 1.5, adjusted P value < 0.05 and variable importance in prediction (VIP) > 1.5). The autophagy-lysosomal pathway was found to be associated with gouty knee arthritis. Essential molecular alterations of multi-omics networks in gouty knee arthritis patients compared with normal controls involve acute inflammatory response, exosomes, immune responses, lysosomes, linoleic acid metabolism, and synthesis.

**Conclusions:**

Comprehensive analysis of proteomic and untargeted metabolomics revealed protein and characteristic metabolite alterations in gouty arthritis, it mainly involves lipids and lipid like molecules, phospholipase A2 and autophagic lysosomes. This study describes the pathological characteristics, pathways, potential predictors and treatment goals of gouty knee arthritis.

**Supplementary Information:**

The online version contains supplementary material available at 10.1186/s12967-023-04114-6.

## Introduction

Acute gout arthritis (AGA) is a inflammatory arthritis caused by the deposition of MSU crystals [1–3]. When the concentration of uric acid (UA) in the blood is higher than 420 μmol/L, it is the first stage of GA. MSU crystals can accumulate in the joint capsule, cartilage, bone, or other periarticular tissues, stimulate the joint synovium and produce pathological responses such as synovial vasodilation, increased permeability and leukocyte exudates [3]. At this point, combined with joint swelling and synovial fluid polarized light microscopy, the patient can be diagnosed with acute gouty arthritis (AGA). Clinically, AGA is most common in major joints, especially the first metatarsophalangeal, ankle, and foot joints. AGA will enter the intermittent symptomatic acute gouty arthritis (ISAGA) phase after uric acid lowering therapy (ULT) or resolve spontaneously [4, 5]. It affects up to 1–2% of adults and is the most common form of inflammatory arthritis in men. It is well known that hyperuricemia and gouty knee arthritis are metabolic diseases. Still, it is uncertain what the metabolic difference between them is and whether this difference contributes to the acute onset of gouty arthritis [6]. At the same time, whether the difference of metabolites is affected by protein expression. Excessive fat intake can lead to the progression of the metabolic disease through cellular damage and inflammation, a process known as lipotoxicity [7, 8].

Autophagy is a critical cellular mechanism for maintaining cellular integrity and has also been implicated in regulating innate immune function [9]. Various effects of excessive lipids and lipid-like molecule accumulation on autophagy have been reported to depend on the tissue under consideration [10]. Here, we investigated the role of lysosomal dysfunction and impaired autophagic flux in the pathogenesis of gouty arthritis. Autophagic degradation activity gradually stalled in knee joints stimulated with urate crystals, associated with impaired lysosomal acidification and excessive lipid accumulation [11]. Previous experimental studies have shown that the accumulated lipids originate from cell membranes [12]. Therefore, it is crucial to know the metabolic-related indicators of gout and the protein expression of related functions [8]. Changes in metabolite and protein expression may contribute to understanding gout susceptibility and its importance for anti-inflammatory pain relief in gouty arthritis. MSU crystals are endogenous danger signals that activate joint-resident cells of the monocyte/macrophage lineage, triggering an inflammatory attack. Inflammation of the synovium produces a large amount of synovial fluid, and many pathological mechanisms of gouty arthritis can be found in the synovial fluid [13]. Although pro-inflammatory cytokines and chemokines are associated with the early stages of acute gouty arthritis, increasing experimental and clinical evidence suggests that autophagy-lysosomes play a vital role in the development of inflammation [10–12]. Activation of autophagy-lysosomes by MSU crystals is thought to regulate inflammatory processes during gout [14].

Lysosomes are membrane-bound organelles containing acid hydrolases involved in the catabolism of intracellular biomolecules and organelles, as well as the catabolism of extracellular components delivered by autophagy endosomal pathways, respectively [15, 16]. Lysosomes also play a crucial role in cell membrane repair and are platforms for cell signalling, making them essential for maintaining stable intracellular homeostasis [17]. Conversely, defects in lysosomal function, and significantly increased lysosomal membrane permeability (LMP), have been demonstrated in various autoimmune diseases and ageing. Preserving the integrity of the lysosomal membrane is critical for maintaining the lysosomal function and protecting cellular components from lysosomal luminal enzymes [18, 19]. However, the mechanism by which lysosomal lipid membranes are altered under pathological conditions remains unclear.

Autophagy is divided into three categories: macroautophagy, microautophagy, and molecular chaperone-mediated autophagy [20]. It plays an important role in maintaining the homeostasis of the intracellular environment by encapsulating and transporting the substances to be degraded from the autophagosome to the autophagolysosome. The mechanism and process of autophagy: It refers to the formation of autophagosome by the bilayer membrane that falls off from the ribosome-free region of the rough endoplasmic reticulum and contains degraded organelles, proteins and other components in the cell, and then fuses with lysosomes to form autophagosome, which degrades its contained contents to meet the metabolic needs of the cell itself and renew some organelles [21]. During the autophagy process of ingested substances, part or all of the organelles or substances to be degraded are wrapped in a double-layered membrane structure to form autophagosomes [21, 22]. 

Like other organelles, lysosomes are surrounded by a membrane containing phospholipids, making them susceptible to phospholipase activation [23]. Three major phospholipases are present in the joint, namely PLA2G2A/sPLA2 (phospholipase A2, group IIA), PLA2G4A/cPLA2 (phospholipase A2, group IVA [cytosolic, calcium-dependent]) and PLA2G6/iPLA2 (phospholipase A2, group IVA) A2, Group VI). PLA2G2A cleaves fatty acyl bonds at the sn-2 position of glycerophospholipids, releasing mainly arachidonic acid and leaving lysophospholipids in the membrane. Arachidonic acid is then converted to eicosanoid prostanoids, leukotrienes, thromboxane, and other signalling lipid metabolites that may trigger inflammatory responses [24]. Lysophospholipids remaining in the membrane can alter the fluidity and permeability of the membrane [19]. Although the function of the signalling metabolites generated by PLA2G2A has been extensively studied, its impact on the properties of native membranes and the function of the organelles they contain is unknown. It has been reported that under pathological conditions, oxygenated cardiolipin is hydrolyzed on the mitochondrial membrane and attributed to the activation of PLA2G2A. IL-6-mediated activation of PLA2G2A is also thought to contribute to the loss of mitochondrial membrane potential [23, 25]. However, phospholipids are uniquely enriched in mitochondria and largely absent in other cell membranes. The effect of PLA2G4A on the lipid composition of cardiolipin-free cell membranes, such as lysosomes, and how this alteration affects membrane integrity and organelle function has not been elucidated.

The is located in the joint cavity and is the ultrafiltrate of plasma. Plasma penetrates blood vessel wall. It enters the extracellular space and mixes with hyaluronic acid secreted by lining cells on the synovial surface, Constitute clear joint fluid [26]. Arthrofluid examination for diagnosis or differential diagnosis of various joints disease is of great value. Joint fluid examination is particularly important for the diagnosis of monoarthritis [27].

We investigated synovial fluid metabolomics and proteomics to identify differential proteins and metabolites, performed enrichment analysis, and found that lysosomal dysfunction and impaired autophagic flux are involved in the pathogenesis of the gouty arthritis effect. Autophagic degradation activity gradually stalled in knee joints stimulated with urate crystals, associated with impaired lysosomal acidification and excessive lipid accumulation. Previous experimental studies have shown that the accumulated lipids originate from cell membranes. Therefore, it is vital to know the metabolic-related indicators of gout and the protein expression of related functions. Differential proteins and differential metabolites may help to understand susceptibility to gout and its importance for anti-inflammatory and pain relief for gouty arthritis. Here, we demonstrate synovial lysosomal membrane permeability (LMP) in gouty arthritis and use synovial fluid metabolomics and proteomics to show that this effect is mediated through the activation of PLA2G2A. Our data demonstrate that PLA2G2A-mediated LMP can release lysosomal enzymes into the cytoplasm, inhibiting autophagic flux and immune cell damage in vitro and in vivo. Finally, our data demonstrate that PLA2G2A is involved in LMP and autophagy inhibition in gouty knee arthritis, suggesting that this mechanism may lead to lysosomal and autophagy defects in other immune diseases.

## Methods

### Ethics

The experiments were approved by our University Ethics Committee (Ethics Committee on Biomedical Research, West China Hospital of Sichuan University No.125 2020-(921)).

### Biological samples

Ten patients with gouty arthritis of the knee who were admitted to our hospital from December 1, 2019, to May 30, 2020, and met the inclusion criteria were included. The clinical features, imaging changes, and arthroscopic manifestations of the patients with knee arthritis were retrospectively analyzed. Synovial fluid was collected during the arthroscopic procedure as sample. And 10 cases of relatively normal synovial fluid were selected as the control group at the same time. The general clinical and demographic data of the gouty arthritis patient group (T) and relatively normal group (N) are shown in Table 1. All patients were hospitalized. Acute episodes of gouty knee arthritis lasted no less than 3 days and the duration of GA was more than 1 year. Of the 10 patients with GA (mean age value = 42.3 ± 9.202), 10 (100%) were male. The mean BMI (27.35 ± 1.32 vs. 22.13 ± 2.33 kg/m^2^; p < 0.001) and uric acid (509.4 ± 114.5 vs. 316.2 ± 100.9; p < 0.001) were higher than in the control group. Also, the proportion of GA patients with smoking and drinking habits was much higher than that of healthy volunteers (smokers: 10% vs. 0%; drinkers: 10% vs. 0%). Serum biochemical parameters, including white blood cells (WBC), C-reactive protein (CRP), uric acid (UA), high/low-density lipoprotein (HDL/LDL), total cholesterol (TC) and triglycerides (TG), sedimentation (ESR), absolute monocyte count (MO#) and absolute lymphocyte count (LY#), were measured in all patients using a fully automated serum biochemistry analysis. In surgical removal of gout stones, all patients with gouty knee arthritis were ensured that urate crystals were removed.

**Table 1:** Population and clinical characteristics of patients with gouty arthritis and normal subjects

	T (gouty arthritis)	N (normal group)	*P value*
N	10	10	
Age(years)	42.3 ± 9.202	38.4 ± 8.884	0.159
Male, gender	100%	80%	0.136
Smoker/non-smoker	10%	0%	0.305
Alcohol consumption (%)	10%	0%	0.305
BMI(kg/m2)	27.35 ± 1.32	22.13 ± 2.33	< 0.001
Activity limitations	80%	0%	0
Swollen joints1YES, 2NO	100%	0%	0
Arthrohydrops1YES, 2NO	50%	0%	0.051
Diabetes DM	20%	0%	0.136
Hypertension	20%	0%	0.136
C-reactive protein, CRP mg/L (< 5)	17.7 (3.57–87.68)	1.31 (1.06–1.79)	0.000**
Uric acid (240–490) μmol/L	509.4 ± 114.5	316.2 ± 100.9	0.001*
HDL-cholesterol (mmol/L) (> 0.9)	0.99 ± 0.22	1.37 ± 0.25	0.002*
LDL-cholesterol(mmol/L)(< 0.40)	2.83 ± 1.27	2.56 ± 0.73	0.577*
Cholesterol CHO(mmol/L) (2.8–5.7)	4.51 ± 1.60	4.27 ± 0.81	0.684*
Triglyceride TG(mmol/L)(0.29–1.83)	1.7 (1.37–2.60)	0.74 (0.58–2.00)	0.043**
ESR (< 21)	19.5 (6.5–60.75)	7.00 (3.75–13.75)	0.035**
WBC (3.5–9.5)	7.05 ± 2.79	5.39 ± 0.99	0.093*
MO# (0.1–0.6)	0.47 ± 0.22	0.32 ± 0.05	0.059*
LY# (1.1–3.2)	1.37 (1.26–2.11)	2.05 (1.81–2.31)	0.105**

^*^p-values represent differences among groups as compared by Student’s t-test

^**^p-values represent differences among groups as compared by the Wilcoxon signed-rank test

^***^p-values represent differences among groups as compared by Chi-squared test

### Proteomics

#### Proteomics protein extraction

Take 40 all of the joint solutions per sample and dilute with 400 μL of Binding Buffer (kit: Binding Buffer). Add 850 μL of Binding Buffer and allow it to flow through the column by gravity for activation. Add the diluted sample and allow it to flow gravitationally through the column. Rewash the column again with 600 μL of binding buffer to collect the eluted components from the previous three steps, i.e., the sample after albumin/IgG removal is vacuum freeze-dried. The freeze-dried sample was added to the solution and centrifuged at room temperature at 12,000 × g for 10 min, after which the supernatant was collected and centrifuged again. The supernatant was the total protein solution of the sample.

Determination of protein concentration: Calculate the standard curve based on the standard protein solution's known concentration and absorbance value and calculate the protein concentration value.

Trypsin digestion: 50 μg of protein is taken from each sample according to the measured protein concentration. DTT is added to the above protein solution to a final concentration of 4.5 mM, and incubated at 55 °C for 30 min. The same volume of iodoacetamide was then added to the solution. Add 6 times the volume of acetone to the above solution to precipitate the protein. Collecting the precipitate. Add 100 μL TEAB2 to re-solubilize the deposit. Add 1 mg/ml of Trypsin-TPCK by mass. Terminate the enzymatic reaction by adding phosphoric acid to adjust the pH is 3. The peptides were desalted using a SOLA^™^ SPE 96-well plate. The spectra output from the mass spectrometry were matched with the theoretical spectra generated by the fasta library to transform the machine signals into peptide and protein sequence information and then combined with the sequence information, peptide retention time, and fragment ion information to build the spectral library.

LC–MS/MS high resolution mass spectrometric detection. All samples after enzymatic digestion were mixed with equal amounts of peptides and fractions were separated in the mobile phase at pH = 10 using an Agilent 1100 HPLC system. Separation conditions Chromatographic column: Agilent Zorbax Extend—C18 narrow bore column, 1 × 150 mm, 5 μm. Detection wavelengths: UV 210 nm and 280 nm. mobile phase A: ACN-H2O (2:98, v/v), mobile phase B: ACN-H2O (90:10, v/v Gradient elution conditions: 0–10 min, 2% B; 10–10.01 min, 2–5% B; 10.01–37 min, 5–20% B; 37–48 min, 20–40% B; 48–48.01 min, 40–90% B; The fractions were collected at one-minute intervals from the 10th minute onwards in the order of 1–10 centrifuge tubes. A total of 10 fractions were collected, vacuum freeze-dried, and dried, and the samples were stored frozen for mass spectrometry. Before mass spectrometry injection, each sample was mixed at a volume ratio of iRT: sample to be tested = 1:10 and used as an internal standard. The enzymatically digested peptides of each sample were acquired separately on the machine, and the scan range was set to 350–1250 m/z with an isolation window of 26 m/z. The spectra output from the mass spectrometry were matched to the theoretical spectra generated by the FASTA library to convert the machine signals into peptide and protein sequence information and then combined with the sequence information, peptide retention time, and fragment ion information to build a library of spectra for DIA analysis. Processing of the raw DIA data was done using Spectronaut Pulsar software.

### Metabolomics

Remove samples stored at −80 °C, remove 100 μL of the sample, and add 10 μL each of internal standard (L-2-chlorophenyl alanine, 0.3 mg/mL; Lyso PC17:0, 0.01 mg/mL, both in methanol) and vortex for 10 s; Add 300 μL of precipitant protein methanol–acetonitrile (V: V = 2:1) and vortex for 1 min; Vortex and shake for 1 min; Extract by sonication in an ice-water bath for 10 min; Stand for 30 min at −20 °C; Centrifuge for 10 min (13000 rpm, 4 °C), evaporate 300 μL of supernatant, then re-dissolve with 200 μL of methanol–water (V: V = 1:4), vortex for 30 s and sonicate for 2 min. Centrifuge for 10 min (13,000 rpm, 4 °C). Aspirate 150 μL of supernatant using a syringe, filter using a 0.22 μm organic phase pinhole filter, and transfer to an LC injection vial and store at −80 °C until LC–MS analysis is performed. Quality control samples (QC) were prepared by mixing equal extracts from all samples, with each QC volume being the same as the sample. Metabolic analysis was performed using a liquid mass spectrometry system consisting of an ACQUITY UPLC ultra-performance liquid tandem AB Triple TOF 5600 high-resolution mass spectrometers.

### Western blot

We used the same method as before to perform Western blotting [28]. The primary antibodies we used were anti- LC3B (1:1000, Abclonal, A7198); anti- SQSTM1/p62 (1: 1000, Abclonal, A11483); anti- PLA2G2A (1:1000, Affinity Biosciences, DF6366); BeyoECL Plus (Beyotime, Beijing, China) was used for developing immunoblots, and a Tanon 2500RGel Imaging System (Tanon, Shanghai, China) was used to take pictures and store protein bands (n = 3).

### Correlation networks

Based on protein expression and metabolite response strength data, pearson correlation algorithm is used to calculate the correlation between protein expression and metabolite response strength data. Pearson correlation coefficient r > 0.6 or r < −0.6 is used to construct correlation network in r software environment, and then visualization using Cytoscape.

### Weighted gene co-expression network analysis

There were 979 genes and 20 samples in the original data from the differential proteins in proteomics. The genes with low expression fluctuation (standard deviation ≤ 0.5) were filtered; the remaining 862 genes were 20 samples. Set the power value from 1 to 30, and calculate the corresponding correlation coefficient and the network's average connectivity, respectively. A weighted co-expression network model was established based on the selected power values, and the 862 genes were finally divided into 9 modules. We used the WGCNA package of R language to complete the data analysis and data visualization, and R language and Python were used to complete the data visualization. Pearson correlation algorithm was used to calculate the correlation coefficient and p-value between characteristic genes and traits of modules. The absolute value of the correlation coefficient was greater than or equal to 0.3, and p-value was less than 0.05 as the threshold value, and the modules related to each trait were screened. For each trait-related module, the correlation between module Gene expression and corresponding trait Gene Significance (GS) was calculated, the correlation between module gene expression and Eigengene was calculated, and the correlation analysis of module trait was constructed according to the correlation.

### Statistics

All data are expressed as mean ± S.E.M. Statistical significance between groups was analyzed using a t-test or one-way ANOV a followed by a Tukey post hoc test to correct for multiple comparisons in GraphPad Prism 5 (San Diego, USA). p < 0.05 were considered statistically significant. An online tool based on R scripts, MetaboAnalyst 2.0 was used. Univariate statistical analysis: The Wilcoxon rank sum test and FC were used in this study to analyze the quantitative changes in metabolomic data. Final results were plotted on volcano plots at a difference multiple ≥ 1.5 and p < 0.05. P-values were corrected due to multiple simultaneous extensive hypothesis testing. Confidence protein: The null value in the data matrix is replaced by half of the minimum value, and the data is normalized by the normalize.quantiles function in R package'preprocessCore' after being processed by log2. Multivariate statistical analysis: PCA analysis was first used to observe and evaluate the overall distribution of all samples, and then comparative analysis of the data across groups was attempted in a supervised manner through PLS-DA modeling. OPLS-DA can better distinguish the differences between two groups of metabolites, mainly by orthogonalizing the samples in both groups. Differential metabolites were further screened with VIP > 1. To prevent overfitting of the constructed model, this study used round-robin interaction validation and ranking tests to judge the credibility of the modeling. The R2Y and Q2 obtained from the cross-loop validation were used to visually evaluate the quality of the model construction, and the response ranking test was used to evaluate the accuracy of the OPLS-DA model by random ranking, which was used to exclude bias caused by over-intervention of the grouped data by supervised learning methods. Orthogonal partial least squares—discriminant analysis OPLS-DA is a supervised statistical method of discriminant analysis. This method is modified on the basis of PLS-DA to filter out the noise irrelevant to the classification information, improve the analytical ability and effectiveness of the model, and maximize the differences between different groups within the model. On the OPLS-DA score chart, there are two principal components, namely, prediction principal component and orthogonal principal component. There is only one prediction principal component and multiple orthogonal principal components. OPLS-DA reflects the maximum difference between groups on t1, so it can directly distinguish the inter-group variation from t1, while the orthogonal principal component reflects the intra-group variation. The two groups of samples have significant differences in OPLS-DA score chart. GO functional enrichment analysis (ORA algorithm), KEGG pathway enrichment analysis (ORA algorithm), and network protein interaction analysis were performed to identify the specific pathways and functions of the differential proteins involved. Open database sources, including the Human Metabolome Database, Gene Ontology Resource, Kyoto Encyclopedia of Genes and Genomes (KEGG) pathway database and MetaboAnalyst, were used to identify metabolic pathways. Upstream regulatory genes of synovial differential proteins were analyzed using IPA software and their activation and repression were predicted. The STRING database (https://cn.string-db.org/) was used to analyze the construction of protein interaction networks for differential proteins.

## Results

### The case for differential protein expression

**Fig. 1:**
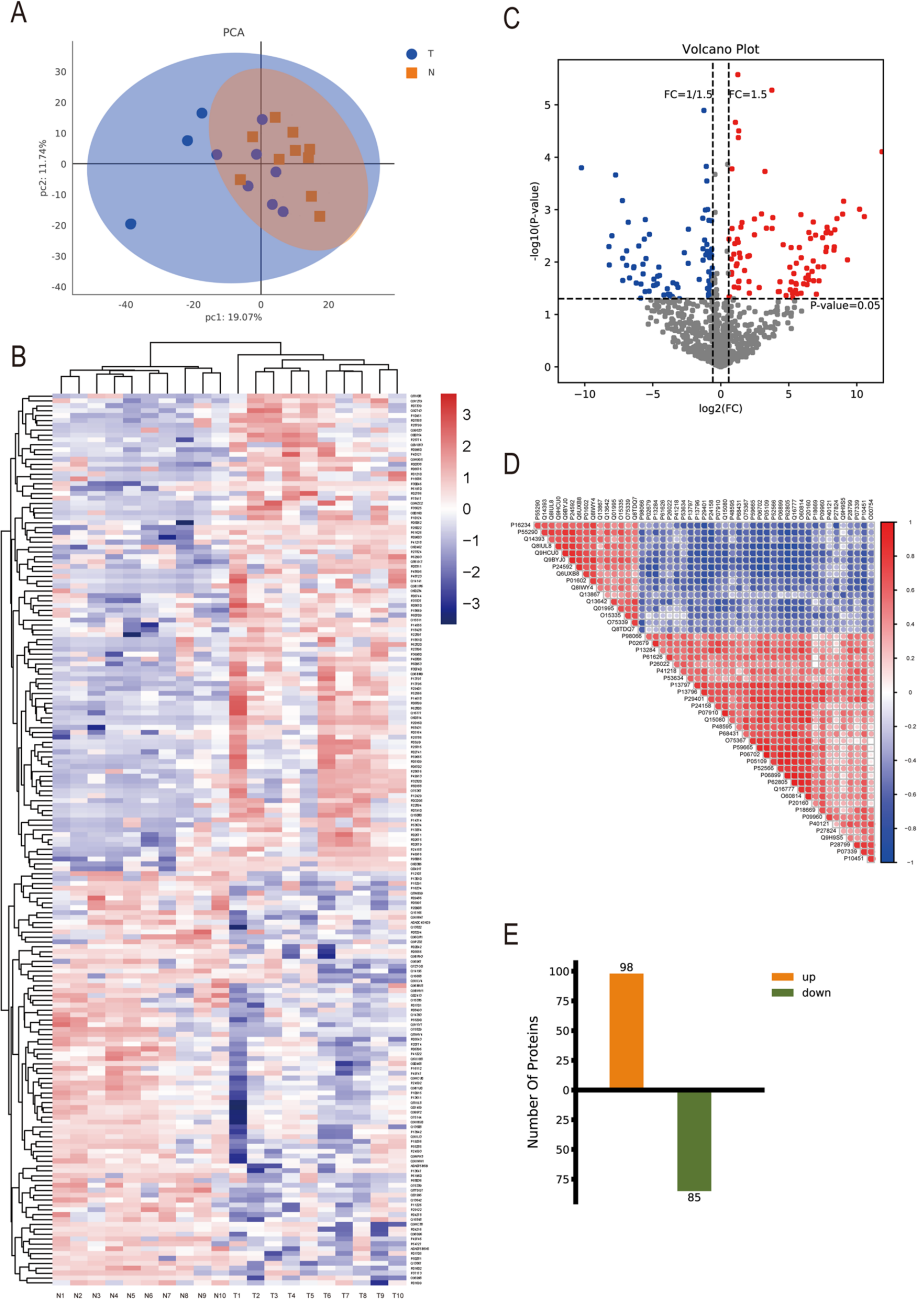
Proteomics of differential protein expression. **A**: Principal component analysis (PCA) using protein expression to show the relationship between samples in different dimensions. Each point in the figure represents a replicate of a grouped experiment, with different colors distinguishing different groups. **B**: Heat map of cluster analysis of differential protein expression comparison groups, with red indicating high expression proteins and blue indicating low expression proteins. Each row represents the expression of each protein in different groups, and each column represents the expression of all differential proteins in each group. The top tree shows the results of cluster analysis of data from different subgroups, and the left tree shows the results of cluster analysis of data from different groups of different proteins. **C**: Volcano plot of differential generation proteins in joint fluid; red represents metabolites up-regulated in group T compared to group N, blue represents down-regulated. **D**: correlation analysis plot of TOP50 differentially significant proteins (pvalue sorted). The graph shows positive correlations in red and negative correlations in blue, the darker the color the greater the correlation. **E**: Statistical plot of differential proteins

Principal component analysis (PCA) was performed using protein expression to show the relationship between samples in different dimensions. Each point represents a replicate of a grouped experiment, and different colors distinguish different groups (Fig. 1A). PCA shows the relationships between samples in different dimensions, with samples in the same group being more spatially distributed.

Clustered heat maps can be used for quality control of standardized experimental data and customized data presentation after differential data enrichment. Data and samples can be clustered to observe sample quality, or co-expression data can be grouped according to expression profiles (Fig. 1B). Attached is a table for converting the gene symbol protein ID (Table 2). In proteomics, the number of trusted proteins expressed was 979 (Additional file 3: Table S1). Unsupervised hierarchical clustering is based on the R language, and in general, samples of the same class can appear in the same cluster by clustering. The red color indicates high expression proteins and the blue color indicates low expression proteins. Each row indicates the expression of each protein in different groups, and each column indicates the expression of all differential proteins in each group. The top tree shows the results of the cluster analysis of the data from the different groups, and the left tree shows the results of the cluster analysis of the data from the different groups for the different proteins. It can be seen that there is a significant difference in protein expression between the gout group and the normal group. The abscissa of the volcanic map is log2 (FC) (Fig. 1C). The farther the value is from the 0 point, the greater the difference. The right side is up, and the left side is down. The ordinate is—log10 (P-value). The farther the ordinate value is from the 0 point, the greater the difference. The blue dots in the figure represent down-regulated differentially expressed proteins, the red dots represent up-regulated differentially expressed proteins, and the gray dots represent non-significant differentially expressed proteins. Correlation analysis of the differential protein expression (Fig. 1D) revealed that the expression of the four histone proteases Cathepsin D, Cathepsin B, Cathepsin G, and Cathepsin S was increased. A total of 183 differential proteins were found, including 98 up-regulated proteins and 85 epitope-sized down-regulated proteins (Fig. 1E, Additional file 4: Table S2).

**Table 2:** the conversions gene symbol-protein ID

Accession	ProteinDescriptions	Genes
A0A075B6I9	Immunoglobulin lambda variable 7–46	IGLV7-46
A0A075B6K5	Immunoglobulin lambda variable 3–9	IGLV3-9
A0A0C4DH29	Immunoglobulin heavy variable 1–3	IGHV1-3
O00468	Agrin	AGRN
O15335	Chondroadherin	CHAD
O75144	ICOS ligand	ICOSLG
O75339	Cartilage intermediate layer protein 1	CILP
O75829	Leukocyte cell-derived chemotaxin 1	CNMD
O95967	EGF-containing fibulin-like extracellular matrix protein 2	EFEMP2
O95998	Interleukin-18-binding protein	IL18BP
P01602	Immunoglobulin kappa variable 1–5	IGKV1-5
P01699	Immunoglobulin lambda variable 1–44	IGLV1-44
P01701	Immunoglobulin lambda variable 1–51	IGLV1-51
P01705	Immunoglobulin lambda variable 2–23	IGLV2-23
P02042	Hemoglobin subunit delta	HBD
P04216	Thy-1 membrane glycoprotein	THY1
P04278	Sex hormone-binding globulin	SHBG
P05543	Thyroxine-binding globulin	SERPINA7
P05556	Integrin beta-1	ITGB1
P05997	Collagen alpha-2(V) chain	COL5A2
P06396	Gelsolin	GSN
P08294	Extracellular superoxide dismutase [Cu–Zn]	SOD3
P08493	Matrix Gla protein	MGP
P09486	SPARC	SPARC
P10915	Hyaluronan and proteoglycan link protein 1	HAPLN1
P11226	Mannose-binding protein C	MBL2
P12107	Collagen alpha-1(XI) chain	COL11A1
P13010	X-ray repair cross-complementing protein 5	XRCC5
P13611	Versican core protein	VCAN
P13647	Keratin, type II cytoskeletal 5	KRT5
P13942	Collagen alpha-2(XI) chain	COL11A2
P15291	Beta-1,4-galactosyltransferase 1	B4GALT1
P16112	Aggrecan core protein	ACAN
P16234	Platelet-derived growth factor receptor alpha	PDGFRA
P19256	Lymphocyte function-associated antigen 3	CD58
P20774	Mimecan	OGN
P20908	Collagen alpha-1(V) chain	COL5A1
P24592	Insulin-like growth factor-binding protein 6	IGFBP6
P24593	Insulin-like growth factor-binding protein 5	IGFBP5
P29122	Proprotein convertase subtilisin/kexin type 6	PCSK6
P37173	TGF-beta receptor type-2	TGFBR2
P41222	Prostaglandin-H2 D-isomerase	PTGDS
P49746	Thrombospondin-3	THBS3
P49747	Cartilage oligomeric matrix protein	COMP
P50281	Matrix metalloproteinase-14	MMP14
P54727	UV excision repair protein RAD23 homolog B	RAD23B
P55285	Cadherin-6	CDH6
P55290	Cadherin-13	CDH13
P61960	Ubiquitin-fold modifier 1	UFM1
P68036	Ubiquitin-conjugating enzyme E2 L3	UBE2L3
Q01459	Di-N-acetylchitobiase	CTBS
Q01995	Transgelin	TAGLN
Q02413	Desmoglein-1	DSG1
Q13508	Ecto-ADP-ribosyltransferase 3	ART3
Q13642	Four and a half LIM domains protein 1	FHL1
Q13822	Ectonucleotide pyrophosphatase/phosphodiesterase family member 2	ENPP2
Q13867	Bleomycin hydrolase	BLMH
Q14195	Dihydropyrimidinase-related protein 3	DPYSL3
Q14393	Growth arrest-specific protein 6	GAS6
Q15166	Serum paraoxonase/lactonase 3	PON3
Q15848	Adiponectin	ADIPOQ
Q16658	Fascin	FSCN1
Q6UXB8	Peptidase inhibitor 16	PI16
Q7Z7G0	Target of Nesh-SH3	ABI3BP
Q8IUL8	Cartilage intermediate layer protein 2	CILP2
Q8IWY4	Signal peptide, CUB and EGF-like domain-containing protein 1	SCUBE1
Q8NBS9	Thioredoxin domain-containing protein 5	TXNDC5
Q8TDQ7	Glucosamine-6-phosphate isomerase 2	GNPDA2
Q96MU8	Kremen protein 1	KREMEN1
Q96QR1	Secretoglobin family 3A member 1	SCGB3A1
Q96S96	Phosphatidylethanolamine-binding protein 4	PEBP4
Q99972	Myocilin	MYOC
Q9BRK3	Matrix remodeling-associated protein 8	MXRA8
Q9BWV1	Brother of CDO	BOC
Q9BYJ0	Fibroblast growth factor-binding protein 2	FGFBP2
Q9HC38	Glyoxalase domain-containing protein 4	GLOD4
Q9HCU0	Endosialin	CD248
Q9NPH3	Interleukin-1 receptor accessory protein	IL1RAP
Q9P232	Contactin-3	CNTN3
Q9UBG0	C-type mannose receptor 2	MRC2
Q9ULI3	Protein HEG homolog 1	HEG1
Q9ULV4	Coronin-1C	CORO1C
Q9UM47	Neurogenic locus notch homolog protein 3	NOTCH3
Q9UNW1	Multiple inositol polyphosphate phosphatase 1	MINPP1
Q9Y5Y7	Lymphatic vessel endothelial hyaluronic acid receptor 1	LYVE1
O00160	Unconventional myosin-If	MYO1F
O00754	Lysosomal alpha-mannosidase	MAN2B1
O15511	Actin-related protein 2/3 complex subunit 5	ARPC5
O60234	Glia maturation factor gamma	GMFG
O60306	RNA helicase aquarius	AQR
O60462	Neuropilin-2	NRP2
O60814	Histone H2B type 1-K	H2BC12
O75367	Core histone macro-H2A.1	H2AFY
P00338	L-lactate dehydrogenase A chain	LDHA
P01210	Proenkephalin-A	PENK
P02671	Fibrinogen alpha chain	FGA
P02675	Fibrinogen beta chain	FGB
P02679	Fibrinogen gamma chain	FGG
P02741	C-reactive protein	CRP
P02786	Transferrin receptor protein 1	TFRC
P05062	Fructose-bisphosphate aldolase B	ALDOB
P05109	Protein S100-A8	S100A8
P05164	Myeloperoxidase	MPO
P06576	ATP synthase subunit beta, mitochondrial	ATP5F1B
P06702	Protein S100-A9	S100A9
P06744	Glucose-6-phosphate isomerase	GPI
P06899	Histone H2B type 1-J	H2BC11
P07339	Cathepsin D	CTSD
P07858	Cathepsin B	CTSB
P07910	Heterogeneous nuclear ribonucleoproteins C1/C2	HNRNPC
P08311	Cathepsin G	CTSG
P08670	Vimentin	VIM
P09603	Macrophage colony-stimulating factor 1	CSF1
P09960	Leukotriene A-4 hydrolase	LTA4H
P0C0S5	Histone H2A.Z	H2AZ1
P0DJI9	Serum amyloid A-2 protein	SAA2
P10124	Serglycin	SRGN
P10451	Osteopontin	SPP1
P12429	Annexin A3	ANXA3
P13284	Gamma-interferon-inducible lysosomal thiol reductase	IFI30
P13796	Plastin-2	LCP1
P13797	Plastin-3	PLS3
P14314	Glucosidase 2 subunit beta	PRKCSH
P14555	Phospholipase A2, membrane associated	PLA2G2A
P14618	Pyruvate kinase PKM	PKM
P15586	N-acetylglucosamine-6-sulfatase	GNS
P18428	Lipopolysaccharide-binding protein	LBP
P18510	Interleukin-1 receptor antagonist protein	IL1RN
P18669	Phosphoglycerate mutase 1	PGAM1
P20160	Azurocidin	AZU1
P22894	Neutrophil collagenase	MMP8
P22897	Macrophage mannose receptor 1	MRC1
P23396	40S ribosomal protein S3	RPS3
P24158	Myeloblastin	PRTN3
P25774	Cathepsin S	CTSS
P25786	Proteasome subunit alpha type-1	PSMA1
P25815	Protein S100-P	S100P
P26022	Pentraxin-related protein PTX3	PTX3
P27824	Calnexin	CANX
P28676	Grancalcin	GCA
P28799	Progranulin	GRN
P29401	Transketolase	TKT
P30046	D-dopachrome decarboxylase	DDT
P30740	Leukocyte elastase inhibitor	SERPINB1
P32320	Cytidine deaminase	CDA
P35625	Metalloproteinase inhibitor 3	TIMP3
P36980	Complement factor H-related protein 2	CFHR2
P37837	Transaldolase	TALDO1
P40121	Macrophage-capping protein	CAPG
P40306	Proteasome subunit beta type-10	PSMB10
P41218	Myeloid cell nuclear differentiation antigen	MNDA
P46976	Glycogenin-1	GYG1
P48595	Serpin B10	SERPINB10
P48723	Heat shock 70 kDa protein 13	HSPA13
P49913	Cathelicidin antimicrobial peptide	CAMP
P52566	Rho GDP-dissociation inhibitor 2	ARHGDIB
P53634	Dipeptidyl peptidase 1	CTSC
P59665	Neutrophil defensin 1	DEFA1
P60709	Actin, cytoplasmic 1	ACTB
P60953	Cell division control protein 42 homolog	CDC42
P61626	Lysozyme C	LYZ
P61970	Nuclear transport factor 2	NUTF2
P62805	Histone H4	H4C1
P62820	Ras-related protein Rab-1A	RAB1A
P62993	Growth factor receptor-bound protein 2	GRB2
P68431	Histone H3.1	H3C1
P78417	Glutathione S-transferase omega-1	GSTO1
P80188	Neutrophil gelatinase-associated lipocalin	LCN2
P98066	Tumor necrosis factor-inducible gene 6 protein	TNFAIP6
Q04917	14–3-3 protein eta	YWHAH
Q0VD83	Apolipoprotein B receptor	APOBR
Q14141	Septin-6	SEPTIN6
Q15080	Neutrophil cytosol factor 4	NCF4
Q16777	Histone H2A type 2-C	HIST2H2AC
Q86UX7	Fermitin family homolog 3	FERMT3
Q8IV08	5'-3' exonuclease PLD3	PLD3
Q92743	Serine protease HTRA1	HTRA1
Q96BM9	ADP-ribosylation factor-like protein 8A	ARL8A
Q99523	Sortilin	SORT1
Q9BXR6	Complement factor H-related protein 5	CFHR5
Q9H9S5	Fukutin-related protein	FKRP
Q9NZC2	Triggering receptor expressed on myeloid cells 2	TREM2
Q9Y279	V-set and immunoglobulin domain-containing protein 4	VSIG4

### GO/KEGG enrichment analysis of differential proteins to characterize their function

**Fig. 2:**
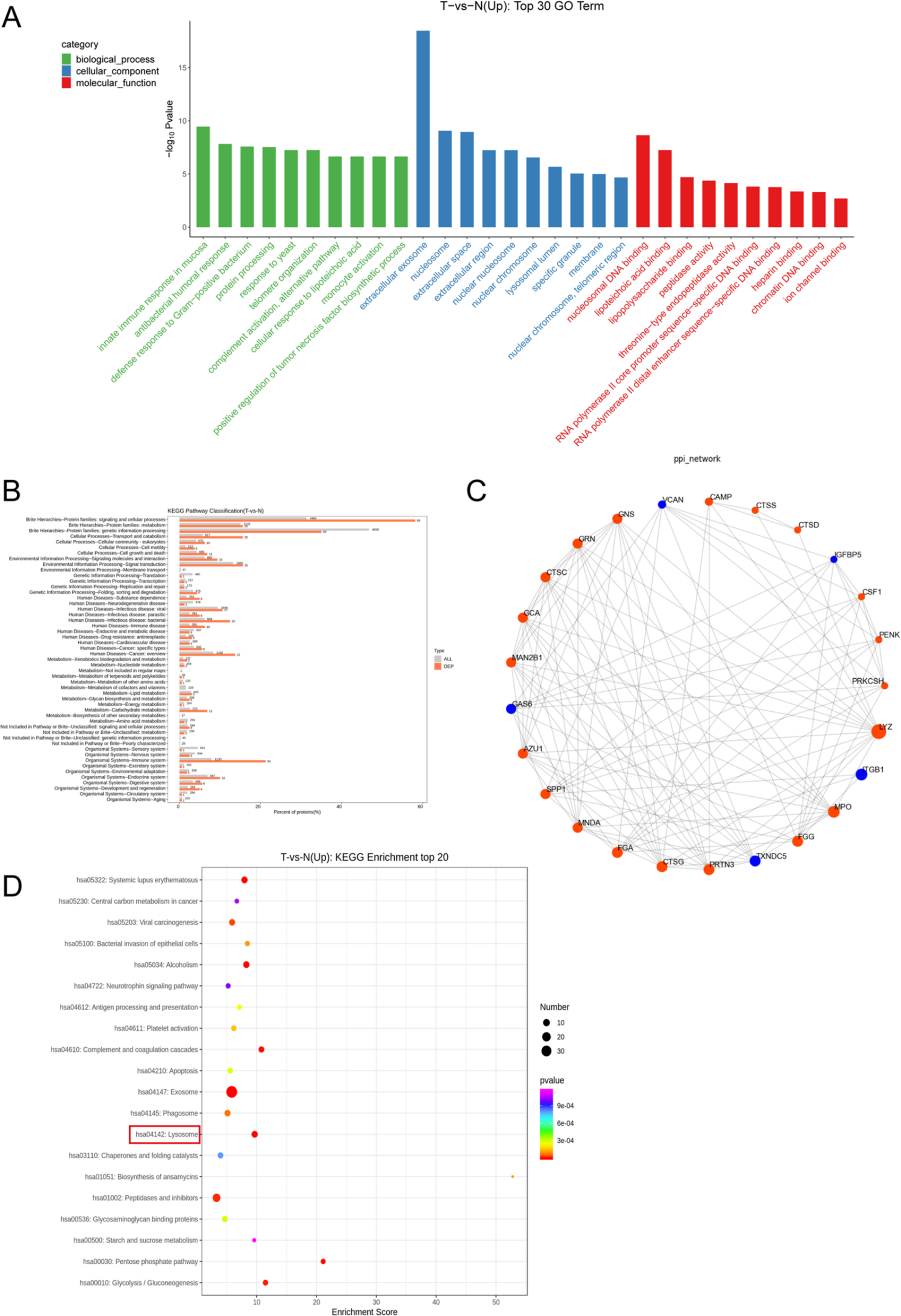
GO/KEGG enrichment analysis of differential proteins to characterize their function. **A**: GO enrichment analysis of elevated expression top30 (screening of 10 GO entries corresponding to the number of proteins greater than 1 in each of the three classifications, sorted by the -log10Pvalue of each entry in descending order), where the x-coordinate is the name of the GO entry and the y-coordinate is the -log10Pvalue.** B**: Comparison of differentially expressed proteins and all proteins at the KEGG Level2 level. Distribution comparison graph. The horizontal axis is the ratio (%) of proteins annotated to each Level2 metabolic pathway (differential proteins) to the total number of all proteins annotated to the KEGG pathway (differential proteins), the vertical axis indicates the name of the Level2 pathway, and the number to the right of the column represents the number of differentially expressed proteins annotated to that Level2 pathway. **C**: PPI protein interaction network, select this species/related species (blast e-value: 1e-10) in STRING database to analyze the differential proteins and obtain the interaction relationship of the differential proteins. **D**: KEGG enrichment analysis top20 (upregulated differential) bubble plot. The x-axis Enrichment Score is the enrichment score and the y-axis is the pathway information of top20. The larger the bubble, the greater the number of differential proteins contained in the entry, and the color of the bubble changes from red–green–blue-violet, the smaller the enrichment p-value and the greater the significance

To gain insight into the biological functions of DEPs, the Gene Ontology (GO) terminology was enriched to include terms for biological processes, cellular components, and molecular functions (Fig. 2A). Thus, DEPs are predicted to be involved in GO terms such as synovial intrinsic immune response, lysosomal lumen and cellular response to lipophilic acid. The most significant cellular components are extracellular associated proteins while synovial intrinsic immune response and complement activation are highlighted in the biological process. Comparison of the distribution of differentially expressed proteins and all proteins at KEGG Level2 level (Fig. 2B) Protein families: metabolism, Immune system, and Endocrine system changes are highlighted. This is why we performed a metabolomic analysis of the synovial fluid in gouty arthritis, identifying relevant metabolite changes and the relationship between proteins and metabolites. To illustrate how DEPs in gouty arthritis are associated with each other, a protein–protein interaction (PPI) network between DEPs was constructed based on the string database (Fig. 2C). Histone protease-related genes with larger sizes in the network were shown to play a more active role in protein–protein interactions associated with gouty arthritis. These include upregulated albumins (Cathepsin B, Cathepsin D, Cathepsin S, Cathepsin G), reflecting the persistent stimulation of synovial membranes by urate crystals, which promotes the production or activation of these proteases, and the positive correlation between them. KEGG pathway enrichment of DEPs Kyoto Encyclopedia of Genes and Genomes (KEGG) Analysis shows that DEPs are mainly enriched in the exosome, lysosomal, and pentose phosphate pathways. Focusing on differentially expressed proteins that are upregulated, the lysosomal pathway was found to be more closely associated with protein changes (Fig. 2D). The lysosomal pathway is shown (Additional file 1: Figure S1), with upregulation of differential protein expression indicated in red.

### Gene co-expression network analysis looks for co-expressed gene modules and explores the association between gene networks and disease phenotypes

**Fig. 3:**
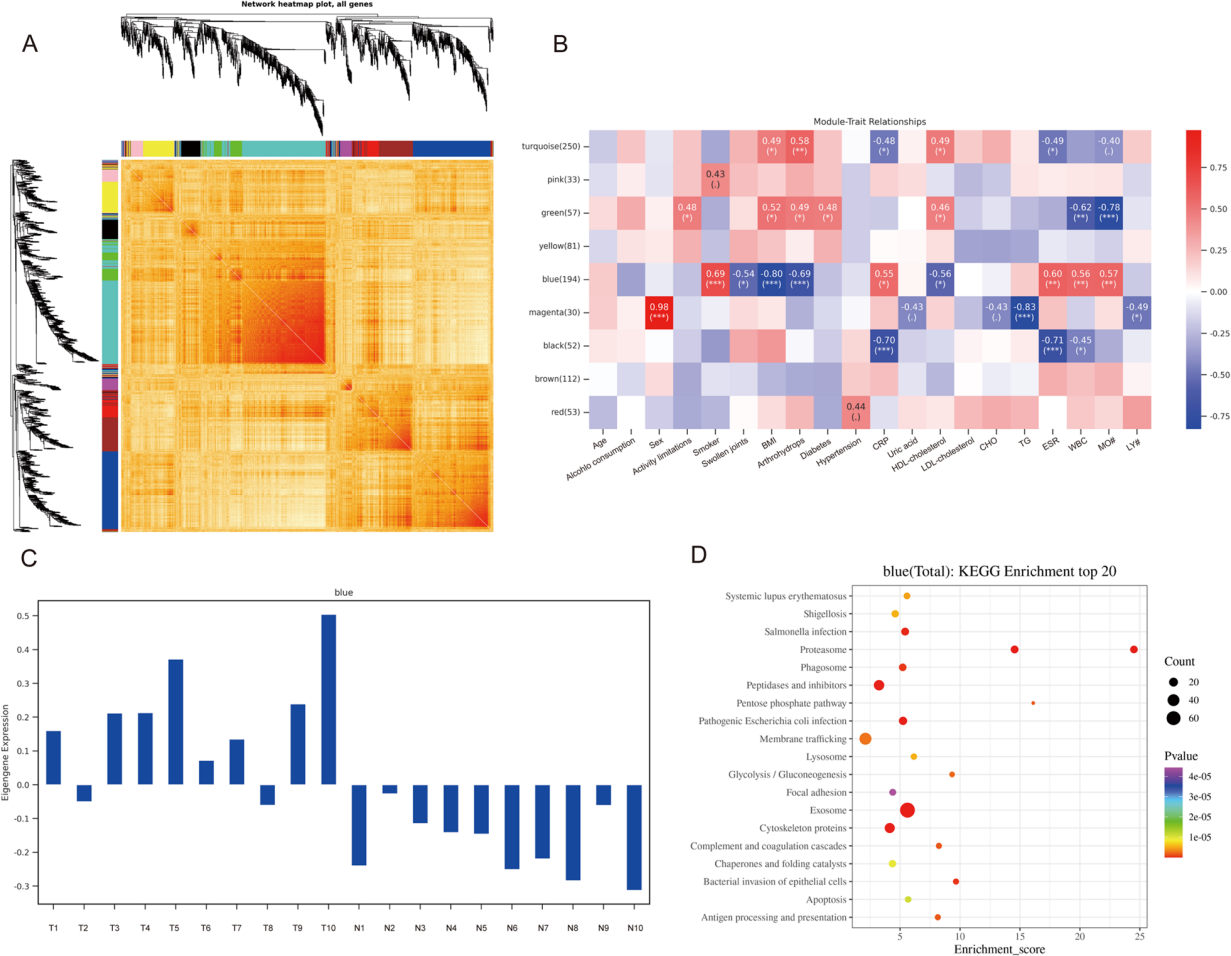
Gene co-expression network analysis looks for co-expressed gene modules and explores the association between gene networks and disease phenotypes. **A**: Heat map of all gene clustering. Based on the selected power values, a weighted co-expression network model was built to finally classify the 862 genes into 9 modules. **B**: Trait module association heat map (absolute value of correlation coefficient greater than or equal to 0.3 and pValue less than 0.05 is the threshold to filter the modules associated with each trait). **C**: Blue module characteristic gene histogram. **D**: KEGG enrichment analysis of genes in the blue module top20 (upregulated differences) bubble plots. Where the x-axis Enrichment Score is the enrichment score and the y-axis is the pathway information for top20. The larger the bubble the more entries contain the number of differential proteins, the bubble color changes from red–green–blue-purple, and the smaller its enrichment p-value, the more significant it is

Based on the selected power values, a weighted co-expression network model was built and the 862 genes were finally classified into 9 modules (Additional file 1: Figure S2). This is the clustering heat map of all genes (Fig. 3A). A list with all the proteins associated with each WGCNA module (Additional file 5: Table S3). In the trait module association heat map (Fig. 3B) we found that the genes in the blue module were positively associated with traits associated with blue,for example Erythrocyte sedimentation rate (ESR), White blood cell count (WBC), Absolute value of monocyte (MO#)). Thus, the blue module trait genes are closely associated with gout attacks. In the blue module signature gene expression information, the expression was found to be increased in the gout group, while the expression of the blue module signature gene was decreased in the normal group (Fig. 3C). Then KEGG pathway enrichment of DEPs for Kyoto genes and genome encyclopedia (KEGG) analysis showed that the face module signature genes were mainly enriched in exosomes, proteasomes, and lysosomes (Fig. 3D). These pathways were positively correlated with the clinical features of gouty arthritis. ESR, WBC and MO# were significantly increased in gouty arthritis patients, while blue module protein showed a correlation. Therefore, in the end, we verified the changes in autophagy flux.

### Metabolomic analysis of synovial fluid in the knee joint of patients with gout and normal group

**Fig. 4:**
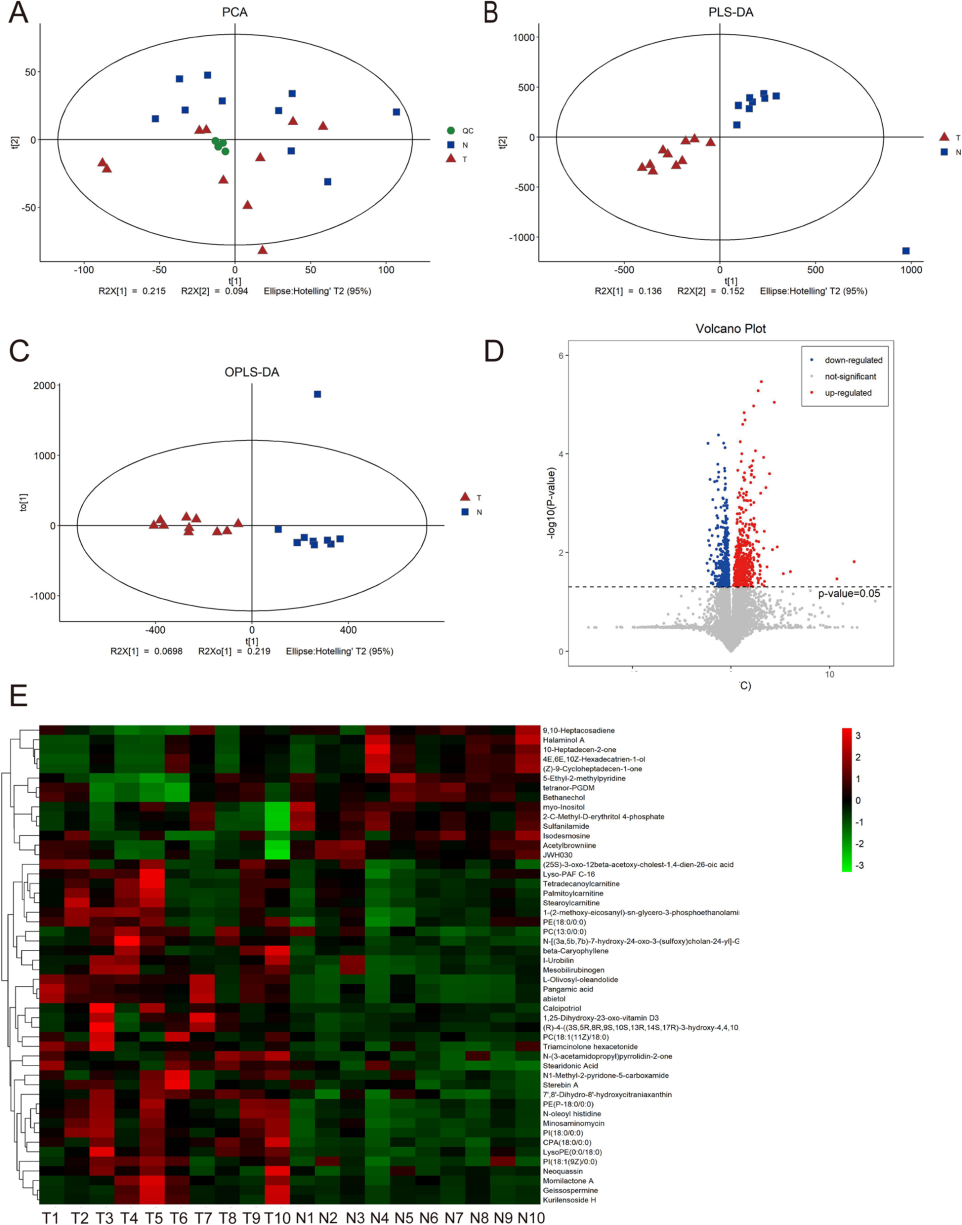
Metabolomic analysis of synovial fluid in the knee joint of patients with gout and normal group. **A**: PCA model plot for all samples obtained by sevenfold cross-validation. **B**: PLS-DA (partial least squares-discriminant analysis PLS-DA is a supervised discriminant statistical method). **C**: Orthogonal partial least squares-discriminant analysis OPLS-DA is a supervised discriminant statistical method in which there is only one predictive principal component and multiple orthogonal principal components. There can be more than one. The between-group variation is maximized on t1 so that between-group variation is directly distinguished from t1, while within-group variation is reflected in the orthogonal principal component. The two groups are significantly different on the OPLS-DA score plot. **D**: Volcanic map can be used to visualize *p* value and Fold change value, which is beneficial to screen differential metabolites. The red origin represents the significantly up-regulated differential metabolites in the experimental group, the blue origin represents the significantly down-regulated differential metabolites, and the gray point represents the insignificant differential metabolites. **E**: TOP-50 differential metabolite heat map, visualization of top50 differential metabolite expression based on VIP values (horizontal coordinates indicate sample names, vertical coordinates indicate differential metabolites. The color ranges from green to red indicating low to high metabolite expression abundance, i.e., redder indicates higher expression abundance of the differential metabolite)

A QC sample principal component analysis was first performed to evaluate the stability of the system, as shown in the figure below (Fig. 4A). The PCA model plots obtained by sevenfold cross-validation (7 cycles of cross-validation), with the QC samples closely clustered together, indicate that this experiment is stable and reproducible. Partial least squares-discriminant analysis PLS-DA is a supervised discriminant statistics method, which uses partial least squares regression to model the relationship between metabolite expression and sample grouping. The explanation rate R2Y (cum) and prediction rate Q2 (cum), both close to 1, indicate that the PLS-DA model can better explain and predict the difference between the two groups of samples, representing good predictive power of the model (Fig. 4B). Orthogonal partial least squares-discriminant analysis (OPLS-DA), a supervised discriminant analysis statistic, showed a significant difference between the two groups of samples on the OPLS-DA score plot (Fig. 4C). One volcano plot for all metabolites (Fig. 4D). We then identified differential metabolites based on VIP values greater than one, with P < 0.05 being significant, for a total of 106 differential metabolites (Additional file 6: Table S4). Hierarchical Clustering was performed to visualize the top50 differential metabolite expressions based on VIP values, and the results are shown in Figure (Fig. 4E), which visualizes the relationship between gout and normal groups and the differences in metabolite expression between samples.

### Enrichment analysis of differential metabolites

**Fig. 5:**
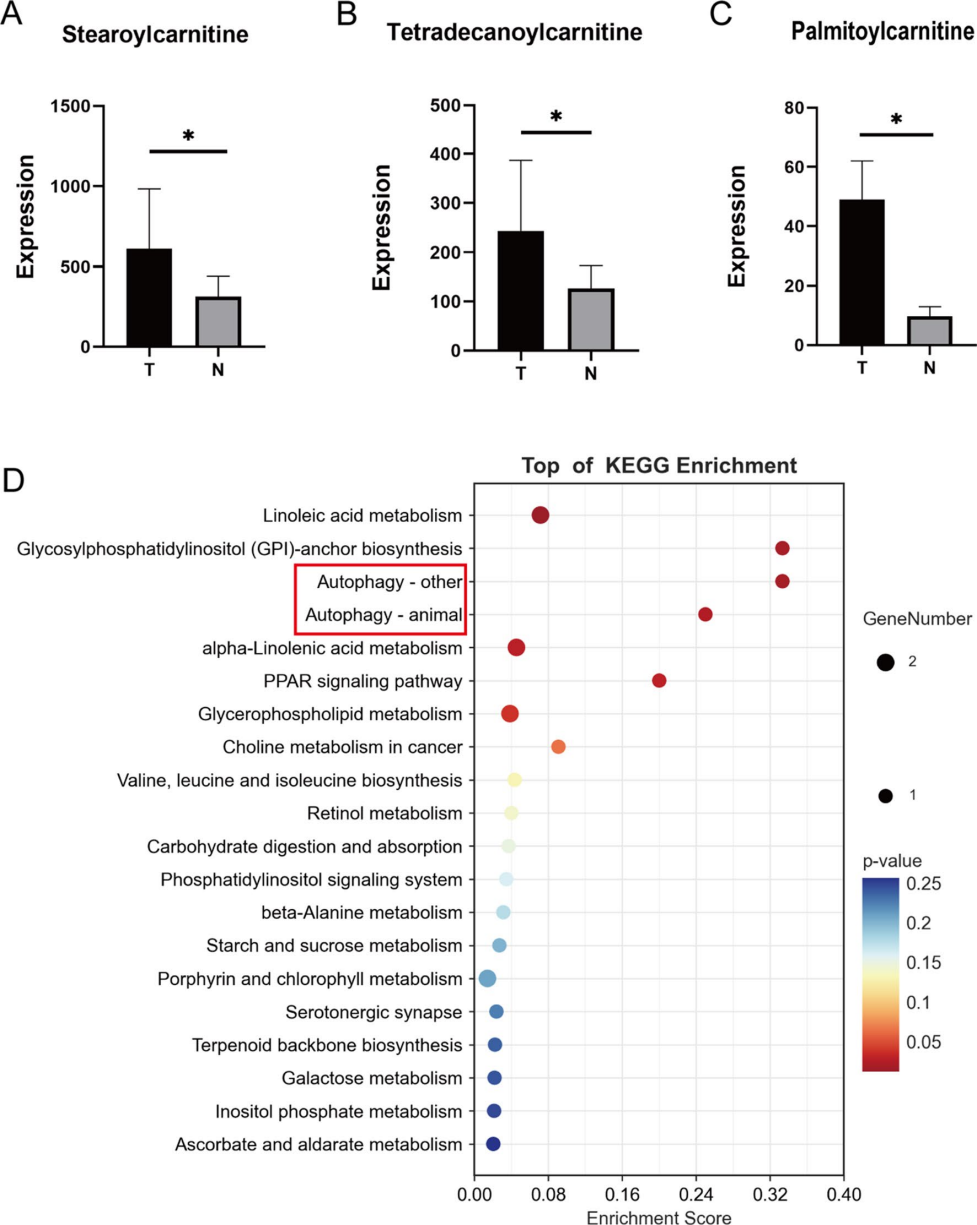
Enrichment analysis of differential metabolites and differential metabolites. **A**, **B** and **C**: Expression of Stearoylcarnitine, Tetradecanoylcarnitine, and Palmitoylcarnitine in joint fluid in each group (n = 10 in each group). * Means p < 0.05, ** means p < 0.01, and *** means p < 0.001. **D**: Bubble plots showing the enrichment of differentially altered pathways. y-axis labels represent pathways, while x-axis labels represent enrichment factors (enrichment factor = number of different metabolites enriched in the pathway). The size and color of the bubbles represent the number of different metabolites enriched in the pathway and the significance of the enrichment, respectively

We found significant differences between lipid and lipid-like molecules such as Stearoyl carnitine, Tetradecanoyl carnitine, and Palmitoylcarnitine (Fig. 5A, B and C) among the differential metabolites. Exploring the pathological changes caused by the significant increase of such metabolites at the disease site requires further biogenic analysis of the significantly increased differential metabolites. The KEGG enrichment analysis of the differential metabolites was also performed, which showed that the differential metabolites were mainly enriched in the Linoleic acid metabolism, Autophagy—other, and Autophagy—animal pathways (Fig. 5D). Therefore, changes in metabolites of lipids and lipid-like molecules as important alterations in gouty arthritis, the increase in associated lipids and lipid-like molecules leads to lip toxicity and thus stimulates an increase in autophagic flux. After reviewing the literature, we selected the autophagic pathway for the next step of validation and exploration.

### Interactions between the proteome and metabolome, and validation of the autophagy pathway

**Fig. 6:**
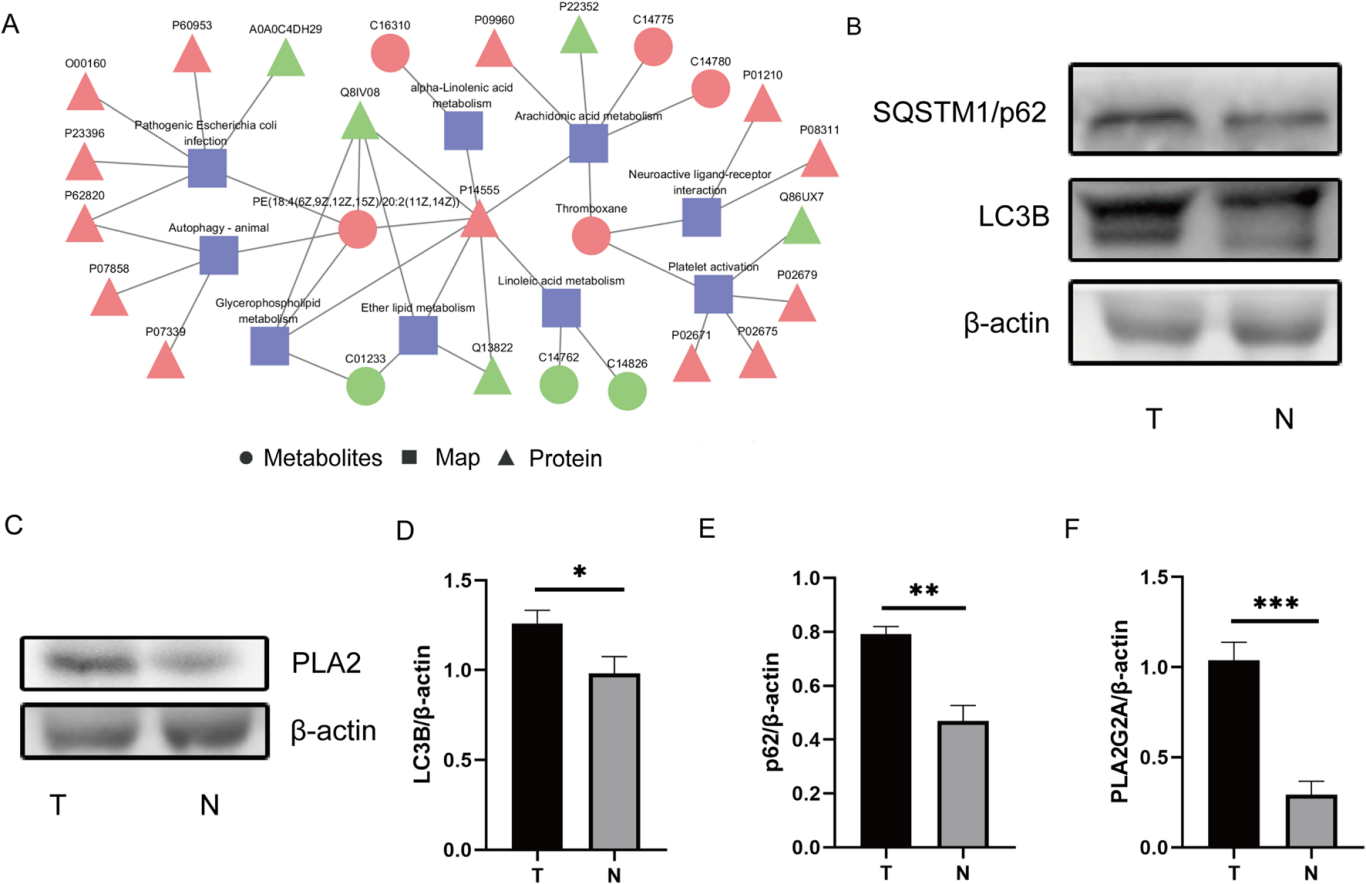
Interactions between the proteome and metabolome, and validation of the autophagy pathway. **A**: Example diagram of protein and metabolism KGML network. Squares represent pathways, triangles represent proteins, and circles represent metabolites. Red indicates up-regulated proteins or green indicates down-regulated proteins or metabolites. **B**: The expressions of LC3B and SQSTM1/62 were evaluated by western blotting. **C**: The expressions of PLA2 were evaluated by western blotting. **D-F:** Quantification of LC3B, SQSTM1/62 and PLA2 expressions (n = 3, all the data are expressed as means ± SD, two-way ANOVA followed by Turkey's post hoc test was applied). * means P < 0.05, ** means P < 0.01, *** means P < 0.001

In the KGML network analysis, it can be found that the protein P14555 (Phospholipase A2) and the metabolite PE are significantly increased, and there is an obvious interaction. At the same time, the autophagy pathway also played a significant role (Fig. 6A). In the verification experiment, Western blot detection of p62 and LC3B showed that the autophagy flux in the T group was higher than that in the N group (Fig. 6B, D and E); the detection of phospholipase A2 showed that the content of the T group was significantly higher than that in the N group (Fig. 6C, E). The autophagic flux obtained from the joint site of gouty knee arthritis was significantly decreased. MSU crystals significantly changed the autophagy pathway under the regulation of phospholipase A2, and many lipids and lipoid substances were found, providing clues to the pathological process of gout arthritis.

## Discussion

A gout is a group of clinical syndromes caused by guanine metabolism disorder or/and reduced uric acid excretion and continuous elevation of serum uric acid, which leads to the precipitation of urate crystals and deposition in tissues or organs [3, 29, 30]. The onset of the population is getting younger and younger, and the population of refractory gout is also increasing [4].The increase of blood uric acid in the human body caused by abnormal purine metabolism is closely related to genetic and environmental factors [30, 31]. The biochemical basis of gout is the increase of blood uric acid in the body. When the blood uric acid concentration reaches a certain saturation, the precipitated urate crystals are deposited in the joints. Kidney and subcutaneous tissue caused by pathological changes, there are various common clinical manifestations of gout, such as arthritis, tophi, and tophi. However, although the number of people with hyperuricemia is huge, about 10% of the people with hyperuricemia suffer from gout [32]. 90% had asymptomatic hyperuricemia and did not progress to the gout stage. The occurrence is strongly related not only to metabolic factors but also to other factors that are not yet known. Clinically visible gout is often accompanied by obesity, diabetes, hypertension, and coronary heart disease, seriously threatening human health [33, 34]. It is bound to harm the development of the national economy [33]. We statistically analyzed the collected 10 gout patients, and the results showed that the patients with gout stones accounted for 100% of the total; The levels of nuclear cells Mo, TG, VLDL, GLU, and uric acid of these patients were significantly increased (P < 0.05 or 0.01), but the levels of HDLC and LDLC were significantly decreased (P < 0.05 or 0.01). The results showed that gout patients were also prone to BMI, blood sugar, blood lipids, and other metabolic abnormalities in addition to elevated inflammatory cells. There have been associated reports of more inflammatory synovial fluid at the joints in obese individuals than in lean patients. For example, the synovial fibroblasts of obese OA patients secrete more IL-6. As a result, there may be other factors in the patient's ventilation that affect the entire course of pathology. In subsequent experiments, we will remove more confounding factors to obtain reliable results and elucidate the pathologies of gouty knee arthritis [35]. Confounding factors include age, sex, and other physical conditions, medication intake which can be adjusted with a larger sample size to ensure the stability of the results. Thus, gout is not only a joint inflammatory disease but a systemic disease that can accumulate in multiple systems. For people with gout, there should also be standardized gout healthy diet, education, promotion of gout hazards and other means, multi-angle management, and diagnosis and treatment of gout. The clinical symptoms of patients with acute gouty arthritis are characterized by significant self-limitation. The self-limiting time in acute gouty arthritis is generally about 1 week, indicating a negative feedback loop regulation in the body. The inflammatory and immune response caused by MSU crystals is currently considered acute gout. Mechanisms of arthritis self-limiting include coating MSU crystals with apolipoprotein B and E and clearing inflammatory cells by macrophages [36, 37]. Some studies have suggested that MSU crystals can activate autophagy and reduce autophagy flux at the basal level, while the Inhibition of autophagy significantly increases the level of IL-1β, suggesting that autophagy may negatively regulate the inflammatory response of gout. ASHFORD TP and PORTER KR. in 1962, who discovered the phenomenon of "self-eating" in cells (material components in cells are degraded by lysosomes) and named its autophagy. Currently, it is believed that acute gouty arthritis is self-limiting [38]. Mechanisms include the coating of MSU crystals with apolipoproteins B and E, the clearance of inflammatory cells by macrophages, the extracellular capture of MSU crystals by neutrophils, and the production of anti-inflammatory cytokines and lipid mediators. Some studies suggest that MSU crystals can activate autophagy and enhance autophagy activity at the basal level, while inhibiting autophagy increases the level of IL-1β, suggesting that autophagy may negatively regulate gout inflammatory responses that are unique to eukaryotic cells [39].

The formation of autophagy-lysosomes is the main feature of autophagy, independent of caspase participation [22]. Not only is autophagy essential in maintaining cell growth, development, differentiation, and homeostasis, but autophagy is also involved in the inflammatory response process. The formation of autophagosomes is related to the autophagy-related genes (Autophagy-related genes, ATG) series genes [21]. LC3 is the homolog of the yeast ATG8 gene present in mammalian cells. LC3 is located on the surface of pre-autophagosome and autophagosome membranes and is involved in the formation of autophagosomes. Currently, LC3 is considered to be the specificity of autophagosomes [40–42]. During the formation of autophagic vacuoles in mammals, LC3 plays a crucial synergistic role in the processing and modification of two ubiquitin-like proteins involved in the synthesis of ATG3, 5, 7, 10, and 12, and plays an essential role in autophagy. After the vesicle is formed, it will continue to bind to the membrane surface of the autophagic vesicle. During autophagosome formation, LC3 binds to phosphatidylethanolamine (PE), a lipid cross-linker that converts soluble LC3 (LC3-I) into an autophagic vesicle-associated form (LC3-II). At this point, the autophagic vesicles are entirely closed to form autophagosomes [43, 44].

At the same time, in metabolomics, we found that the expression of PLA2G2A in the knee joint fluid of gout patients was significantly increased. At the same time, we found that the lipid substances such as Stearoylcarnitine and Tetradecanoylcarnitine were significantly increased. The accumulation of these substances increased, which further led to joint lipotoxicity. Toxic substances and MUS work together to increase local inflammatory response and pain in the knee joint. At the same time, due to the impact on the expression of related proteins and metabolites. Therefore, our omics analysis, through the cluster analysis of differential proteins and differential metabolites, obtained significant differences in related differential proteins phospholipase A2, cathepsin (B, D, G, S); differential metabolites: gouty knee joint Lipids and lipid-like molecules are significantly increased in the synovial fluid of knee joints in patients with arthritis. After enrichment analysis, we found significant changes in the autophagy-lysosome pathway.

Then we verified the correlation analysis results and found that LC3B and p62 in patients with gouty knee arthritis were significantly higher than in regular patients. The results indicated that MSU and lipotoxicity inhibited the autophagy flux in patients with gouty knee arthritis. Our preliminary conclusions are that Stearoylcarnitine, Tetradecanoylcarnitine, Palmitoylcarnitine, and other lipids and lipid-like substances increase the integrity of cell membranes and organelle function, thereby affecting the function of lysosomes and finally, the performance of autophagy-lysosome complexes Restricted, the autophagic flux is reduced. The function of regulating inflammation in the knee joint is lost. The pathological process of gouty arthritis is fundamentally different from that of other arthritis. For example, rheumatoid arthritis is mainly characterized by cell proliferation in the synovial membrane of joints, infiltration of a large number of inflammatory cells in the interstitium, as well as the new formation of microvessel, pannus formation and destruction of cartilage and bone tissue. The synovial cells have tumor phenotype, which is fundamentally different from the metabolic and immune diseases of gouty arthritis [45].

However, this study also faces some problems. Because of the problem of clinical specimen extraction from patients with gouty arthritis, we performed a correlation analysis on the synovial fluid of the patients. We obtained information on differentially expressed proteins and differential metabolites in the synovial fluid of the knee joint. There are some limitations to the existing results. The study had a small sample size due to time and cost. Following an enrichment analysis of proteomics and metabolomics data, we found changes in related protein genes and metabolites that are only preliminary for future studies on the pathological mechanisms of gout-knee arthritis, providing a predictive direction for the pathological changes. The pathogenesis of gouty knee arthritis cannot be unambiguously explained by the validation of changes in autophagy flux. This mechanism needs to be established in animal and in vitro models to fully validate the existing predictions. Further investigations will be carried out in the following studies. In future research, we will explore the metabolic changes of articular cartilage and joint synovium. Due to the limitations of proteomics and metabolomics, in order to further discuss the development mechanism of gouty knee arthritis, further related gene sequencing was used to detect the expression of genes, but genes, proteins, and metabolites, it is not that the influence between them is not a single direction, but the three are mutual. We innovatively explored the interrelationship between proteomics and metabolomics of synovial fluid in gouty knee arthritis linked to the clinical trait module. Found that lipids and lipid-like substances contribute to autophagy- Inhibition of lysosomal complexes interferes with the normal biological functions of related cells, thereby aggravating inflammation and pain in joints, providing specific therapeutic ideas for future clinical diseases. Phospholipase A2, lipid molecules, lysosomal membrane damage, and decreased autophagy flux were found in gouty arthritis patients requiring surgery. These indicators can predict whether other gouty arthritis patients need surgical intervention.

## Supplementary Information

**Additional file 1: Figure S1.** The lysosomal pathway. Rectangle represents enzyme/gene, red represents up-regulated protein, green represents down-regulated protein, and yellow represents both up-regulated and down-regulated corresponding protein. The colorless rectangle represents the genes in the map, the light green rectangle represents the genes unique to the species, and the light purple rectangle represents the genes in both map and ko.

**Additional file 2: Figure S2.** Correlation between seven key modules and clinical traits.

**Additional file 3: Table S1.** In proteomics, the number of trusted proteins expressed was 979.

**Additional file 4: Table S2.** Screening results of differentially expressed proteins in synovial fluid. Two criteria were selected to calculate the difference between samples (T: N). Foldchange is used to assess the fold change in the expression level of a protein between samples; The *p*-value, calculated by t-test, shows the significance of the difference between samples. Foldchange = 1.5 times and p-value < 0.05.

**Additional file 5: Table S3.** A list with all the proteins associated with each WGCNA module.

**Additional file 6: Table S4.** Screening results of different metabolites in synovial fluid. Select two standards to calculate the difference between samples. Foldchange is used to evaluate the change multiple of the expression level of a metabolite among samples (T: N); The p-value calculated by t-test, shows the significance of the difference between samples. Foldchange = 1.5 times and p < 0.05.
