# Supplementary figures and images for: Phospholipase A2 regulates autophagy in gouty arthritis: proteomic and metabolomic studies
Source: J Transl Med. 2023 Apr 17;21:261. doi: 10.1186/s12967-023-04114-6 (PMC10108447; doi:10.1186/s12967-023-04114-6)

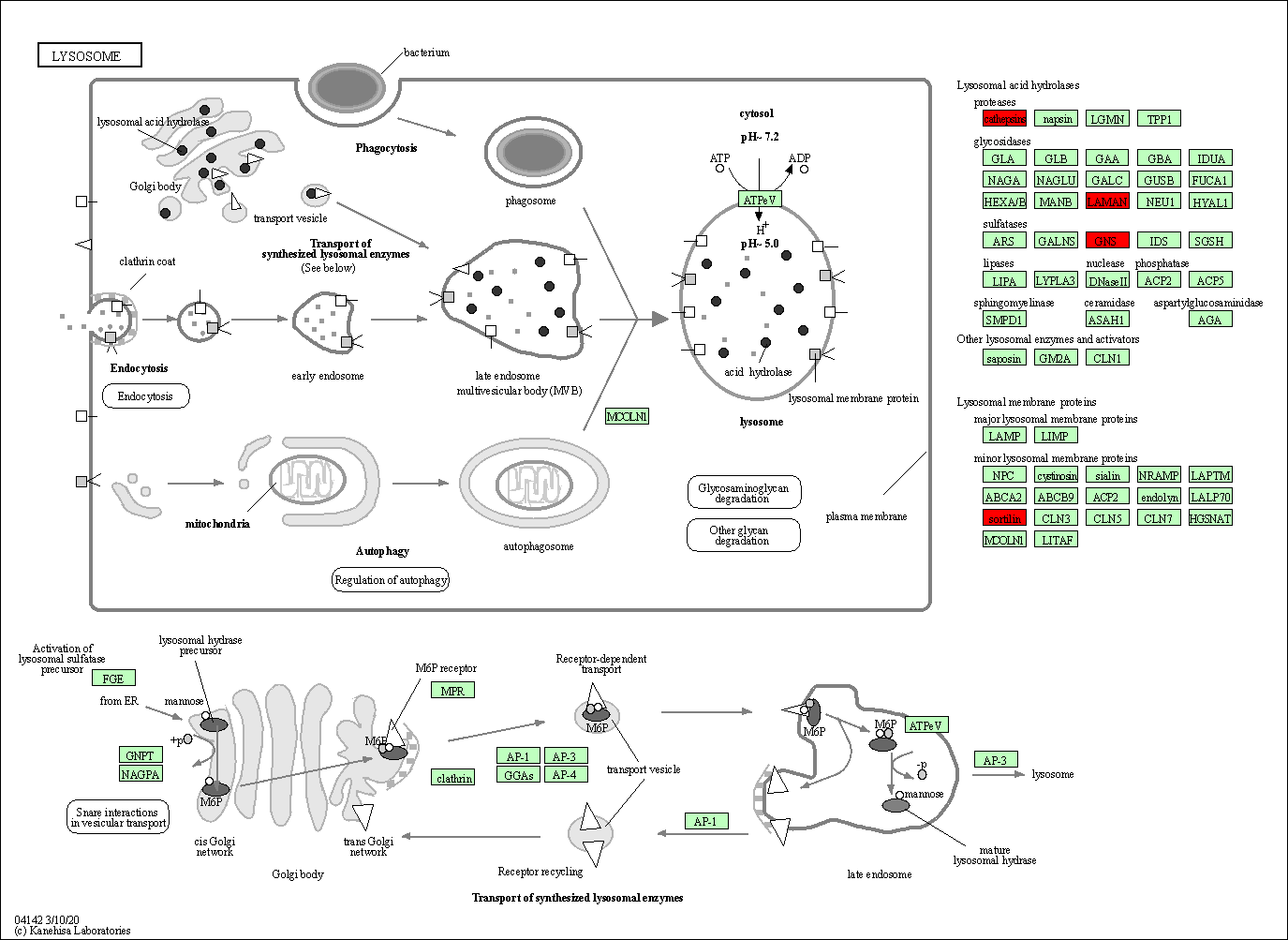

Supplement: Supplementary file 1 — Additional file 1: Figure S1. The lysosomal pathway. Rectangle represents enzyme/gene, red represents up-regulated protein, green represents down-regulated protein, and yellow represents both up-regulated and down-regulated corresponding protein. The colorless rectangle represents the genes in the map, the light green rectangle represents the genes unique to the species, and the light purple rectangle represents the genes in both map and ko. [file 12967_2023_4114_MOESM1_ESM.png]

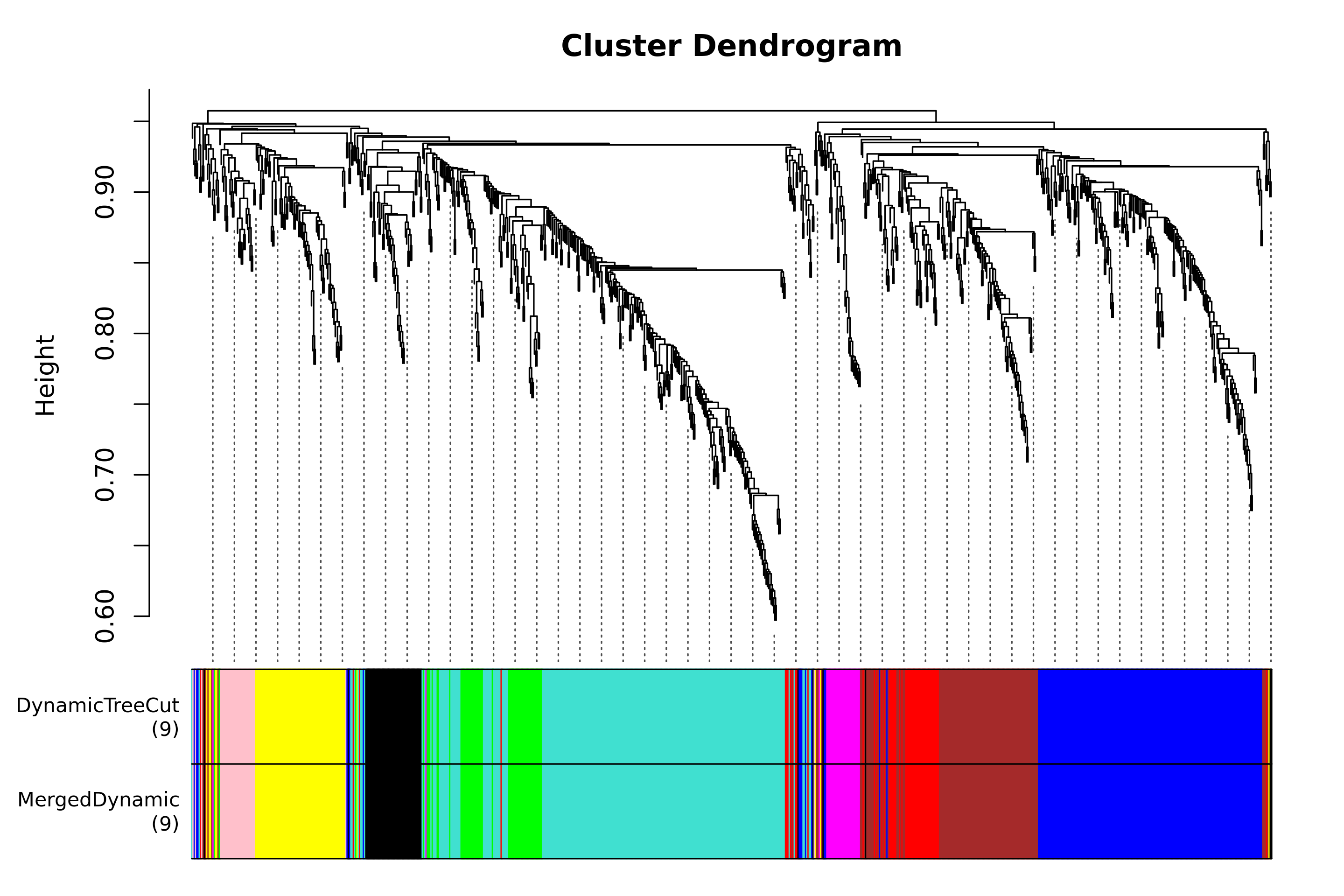

Supplement: Supplementary file 2 — Additional file 2: Figure S2. Correlation between seven key modules and clinical traits. [file 12967_2023_4114_MOESM2_ESM.png]
